# Adipose-derived human stem/stromal cells: comparative organ specific mitochondrial bioenergy profiles

**DOI:** 10.1186/s40064-016-3712-1

**Published:** 2016-12-01

**Authors:** Alice S. Ferng, Katherine M. Marsh, Jamie M. Fleming, Renee F. Conway, David Schipper, Naing Bajaj, Alana M. Connell, Tia Pilikian, Kitsie Johnson, Ray Runyan, Stephen M. Black, John A. Szivek, Zain Khalpey

**Affiliations:** 1 Division of Cardiothoracic Surgery, Department of Surgery, University of Arizona College of Medicine, 1501 North Campbell Avenue, Tucson, AZ 85724 USA; 2 Department of Physiological Sciences, University of Arizona College of Medicine, Tucson, AZ USA; 3 Department of Biomedical Engineering, University of Arizona College of Medicine, Tucson, AZ USA; 4 University of Arizona College of Medicine, Tucson, AZ USA; 5 Department of Orthopaedic Surgery, University of Arizona College of Medicine, Tucson, AZ USA; 6 University of Massachusetts Medical School, UMass Memorial Health Care, Worcester, MA USA; 7 Department of Cellular and Molecular Medicine, University of Arizona College of Medicine, Tucson, AZ USA; 8 Banner-University Medical Center, Tucson, AZ USA

**Keywords:** Adipose-derived stem/stromal cells, Human adipose tissue, Mitochondrial bioenergetics, Bioenergetic profiling, Stromal vascular fraction, Mesenchymal stem cells, Extracellular flux, Tissue engineering

## Abstract

**Background:**

Adipose-derived stem/stromal cells (ASCs) isolated from the stromal vascular fraction are a source of mesenchymal stem cells that have been shown to be beneficial in many regenerative medicine applications. ASCs are an attractive source of stem cells in particular, due to their lack of immunogenicity. This study examines differences between mitochondrial bioenergetic profiles of ASCs isolated from adipose tissue of five peri-organ regions: pericardial, thymic, knee, shoulder, and abdomen.

**Results:**

Flow cytometry showed that the majority of each ASC population isolated from the adipose tissue of 12 donors, with an n = 3 for each tissue type, were positive for MSC markers CD90, CD73, and CD105, and negative for hematopoietic markers CD34, CD11B, CD19, and CD45. Bioenergetic profiles were obtained for ASCs with an n = 4 for each tissue type and graphed together for comparison. Mitochondrial stress tests provided the following measurements: basal respiration rate (measured as oxygen consumption rate [pmol O_2_/min], ATP production, proton leak, maximal respiration, respiratory control ratio, coupling efficiency, and non-mitochondrial respiration. Glycolytic stress tests provided the following measurements: basal glycolysis rate (measured as extracellular acidification rate [mpH/min]), glycolytic capacity, glycolytic reserve, and non-glycolytic acidification.

**Conclusions:**

The main goal of this manuscript was to provide baseline reference values for future experiments and to compare bioenergetic potentials of ASCs isolated from adipose tissue harvested from different anatomical locations. Through an investigation of mitochondrial respiration and glycolysis, it was demonstrated that bioenergetic profiles do not significantly differ by region due to depot-dependent and donor-dependent variability. Thus, although the physiological function, microenvironment and anatomical harvest site may directly affect the characteristics of ASCs isolated from different organ regions, the ultimate utility of ASCs remains independent of the anatomical harvest site.

**Electronic supplementary material:**

The online version of this article (doi:10.1186/s40064-016-3712-1) contains supplementary material, which is available to authorized users.

## Background

Adipose-derived stem/stromal cells (ASCs) isolated from the stromal vascular fraction (SVF) have long been considered an abundant and ideal source of stem cells for tissue regeneration and stem cell research. The SVF is the densest component of the lipoaspirate obtained from liposuction of excess adipose tissue. SVF isolates typically contain many components also found in adipose tissue including adipocytes, fibroblasts, preadipocytes, tissue resident macrophages, and vascular constituents (Baer and Geiger 2012; Aronowitz and Ellenhorn 2013; Gimble et al. 2011). Most importantly, both adipose tissue and SVF provide a source of mesenchymal stem cells (MSCs) that do not elicit an immunological response, particularly if autologous ASCs are used during point-of-care applications (McIntosh et al. 2009; Pikuła et al. 2013). Of the three main sources of MSCs currently used for human studies and therapies—bone marrow, umbilical cord blood, and adipose tissue—ASCs show important differences from bone-marrow derived MSCs in regard to harvest and cell yield, despite similarities in morphology and phenotype. Therefore, ASCs represent an attractive, abundant and readily available cell type for regenerative medicine. ASCs have been successfully used in tissue and wound regeneration such as sternal reconstruction (Khalpey et al. 2015), cosmetic reconstruction (Koźlik and Wójcicki 2014), and chondrogenesis (Mellor et al. 2015; Estes et al. 2010). A major reason ASCs have shown great promise is that ASCs from the SVF have 50–74% of the matrix-forming ability of fibroblasts, which can be helpful in wound healing (Shin et al. 2015).

There are a variety of methods of ASC isolation that include mechanical, chemical, or non-enzymatic techniques. However, collagenase-based isolation methods traditionally yield the highest level of cell recovery. Mechanical methods have reported cell yields ranging from 6250 to 25,000 ASCs/mL of adipose tissue, whereas collagenase methods yield 100,000 to 500,000 viable cells/mL (Aronowitz and Ellenhorn 2013; Shah et al. 2013; Markarian et al. 2014; Aronowitz et al. 2015). While the use of collagenase-isolated ASCs in patient-specific applications may cause injection-related complications such as local allergic reaction, infection, or evidence of local tissue destruction, clinical studies in 164 patients have shown little or no complications related to collagenase isolations (Aronowitz et al. 2015).

ASCs all share similar characteristics despite isolation protocol (Sachs et al. 2012), and it has been recognized that adipocytes demonstrate significant intrinsic inflammatory properties (Omar et al. 2014). Since adipose tissue is a mediator of inflammation and innate immunity, its anatomic location plays a defining role in its specific metabolic functions (Omar et al. 2014; Schwartz and Yehuda-Shnaidman 2014). The bioenergetic demand of cells from various locations is therefore expected to vary, in part due to individual physiologic organ functions. Furthermore, the physiological microenvironment that supports stem cells in specific anatomic locations can regulate how stem cells participate in tissue regeneration, maintenance and repair, and may also be donor-dependent (Via et al. 2012). We therefore hypothesized that bioenergetic demand of adipose tissue will be correlated to intrinsic metabolic function and dependent on anatomical location. In this study, we compare the bioenergetic profiles of freshly isolated adipose tissue of the SVF obtained from the abdomen (subcutaneous), thymus, pericardium, knee (peri-patellar), and shoulder of healthy human patients.

## Methods

### Primary culture of human adipose-derived stem cells

ASCs were isolated from freshly excised human subcutaneous adipose tissue following methods adapted from Estes et al. (2010). Donors (n = 12) were both male and female, between 18 and 65 years of age. All samples were obtained as surgical discards with approval from the University of Arizona Internal Review Board (IRB approval numbers: 01-0770-01 and 1300000194A004) from the adipose tissue surrounding the following regions: abdominal (subcutaneous), knee (peri-patellar), shoulder, pericardium, and thymus. In brief, adipose tissue was minced and washed extensively with 1X PBS + 1% antibiotic–antimycotic (Thermo Scientific, Waltham, MA), and then incubated at 37 °C for 60 min in 2 mg/ml of type I collagenase (Worthington Biochemical, Lakewood, NJ, USA). Enzyme activity was neutralized with Dulbecco’s modified Eagle’s medium–low glucose (DMEM, Gibco, Grand Island, NY, USA), containing 10% FBS, and cells were centrifuged at 1200 rpm for 10 min to remove adipocytes and filtered through a 100 μm nylon mesh to remove debris, and incubated in a culture flask at 37 °C at standard cell culture conditions of 95% O_2_ and 5% CO_2_. Following incubation for 3 days, the cells were washed with 1X PBS to remove non-adherent cells. ASCs were isolated from adipose of 12 donors, with an n = 4 for each tissue type.

### Flow cytometry

Cells extracted from each source were evaluated for mesenchymal stem cell markers (CD90, CD105, and CD73), hematopoietic cell markers (CD34, CD11B, CD19, and CD45), and HLA-DR. Cells were kept between passages 1–3, and analyzed immediately when the cell number required for flow cytometry analysis was achieved in culture.

Culture medium was removed and cells were washed with 1X PBS. Cells were then detached from the flask using BD Accutase Cell Detachment Solution (BD Biosciences, San Jose, CA, Cat #561,527), centrifuged at 1500 rpm for 5 min, and re-suspended in BD Pharmingen stain buffer (BD Biosciences, San Jose, CA, Cat #554656) at a concentration of 5 × 10^6^ cells/mL. The cell suspension was filtered through a 40 μm mesh filter. 100 μL cell suspension was added to each tube containing antibodies from the BD Stemflow Human MSC Analysis Kit (BD Biosciences, San Jose, CA, Cat #562245) and are reported in Table 1. Antibody/cell mixtures were incubated in the dark on ice for 30 min. The tubes were centrifuged at 1500 rpm for 5 min, washed twice with 100 μL stain buffer, and then re-suspended in 500 μL stain buffer.

**Table 1:** Antibodies used for flow cytometry analysis

Tube	Antibody	Flourochrome	Purpose
1	Mouse anti-human CD90	FITC	Compensation
2	Mouse anti-human CD44	PE	Compensation
3	Mouse anti-human CD105	PerCP	Compensation
4	Mouse anti-human CD73	APC	Compensation
5	hMSC positive cocktail and hMSC negative cocktail	FITC (CD90), PerCP (CD015), APC (CD73), PE (CD34, CD11B, CD19, CD45, HLA-DR)	Evaluation of positive and negative MSC markers
6	Unstained	NA	Control
7	hMSC positive isotype control and PE hMSC negative isotype control	FITC (CD90), PerCP (CD015), APC (CD73), PE (CD34, CD11B, CD19, CD45, HLA-DR)	Negative staining control

Cells were analyzed on a FACSCanto™ II equipped with two lasers (blue 488 nm and red 633 nm lasers), six fluorescence channels, and two channels for Forward Scatter and Side Scatter. At least 10,000 gated events were acquired on a log fluorescence scale. After viable and singlet cells were gated, MSCs were first isolated by their positivity for CD90, then by positivity for CD73 and CD105, and were ultimately identified by their negativity for all hematopoietic markers and HLA-DR.

### Bioenergetic profiling

Stress tests were completed using the Seahorse Bioscience XFe96 Flux analyzer according to the manufacturer’s instructions to measure oxygen consumption (mitochondrial stress test) and extracellular acidification (glycolysis stress test). The reagents used for the mitochondrial stress tests were optimized for maximal effect for ASCs isolated from each peri-organ region, and all chemicals were obtained from Seahorse Biosciences or Sigma Aldrich. Cells from passages 3–9 were seeded into 96-well plates at their previously optimized density of 40,000 cells per well and allowed to adhere overnight. Prior to running the mitochondrial or glycolysis stress assays, cells were first washed with assay media containing 3 mM l-glutamine, 1 mM pyruvate and 8 mM glucose, and incubated at 37 °C in a non-CO_2_ environment 60 min prior to the Seahorse assay in order to allow the cells to become equilibrated with the assay medium. The XFe96 sensor cartridge was pre-hydrated with calibrant solution overnight at room temperature prior to the experimental assay.

Oxygen consumption rates (OCR) were measured in the presence of oxidative phosphorylation (OXPHOS) driving substrates. After three basal measurements, three measurements each were taken after the subsequent addition of oligomycin, FCCP (carbonyl cyanide-4-trifluoromethoxyphenylhydrazone), and rotenone/antimycin A combination. These injected drugs block ATP synthase, uncouple the oxygen consumption from ATP synthesis, and block mitochondrial complexes I and III, respectively. For the glycolysis stress test, drugs were similarly optimized for each cell type and injected in the following order: glucose, oligomycin, and 2-DG (2-deoxy-d-glucose; glycolysis inhibitor).

### Mitochondrial stress test calculations

The basal respiration was recorded as the basal OCR minus non-mitochondrial OCR. Following inhibition of ATP synthase by oligomycin, ATP production was calculated by subtracting the proton leak from basal respiration. Proton leak was defined as remaining basal respiration not coupled to ATP production. After injection of the uncoupler FCCP, maximal respiration was calculated by subtracting the non-mitochondrial respiration from the highest OCR value obtained. The respiratory control ratio (RCR) was then calculated by dividing maximal respiration (state 3u) by proton leak (state 4o). Coupling efficiency was calculated as a percentage by dividing ATP production by basal respiration and multiplying the final value by 100.

### Glycolysis stress test calculations

Glycolysis was defined as the extracellular acidification rate (ECAR) due to the utilization of exogenously provided glucose. After addition of glucose, glycolysis was calculated by subtracting non-glycolytic acidification (NGA) ECAR with the highest ECAR value, where NGA was extracellular acidification not due to utilization of exogenous glucose. Following injection of oligomycin, glycolytic capacity was calculated as the basal ECAR subtracted from the highest ECAR used to meet cellular demands after ATP synthase is inhibited. Glycolytic reserve is calculated as a percentage by dividing glycolytic capacity with glycolysis, and multiplying by 100.

### Statistical analysis

A one-way ANOVA post hoc analysis was conducted to compare for significance between each parameter for ASCs isolated from different adipose tissue organ locations. Where there was significance found in the ANOVA test for a measured parameter (α = 0.05), a Tukey’s HSD test was performed to identify pairwise significance. Statistics were performed in R, and graphs were created in GraphPad Prism 6 (La Jolla, CA).

## Results

### Flow cytometry

**Fig. 1:**
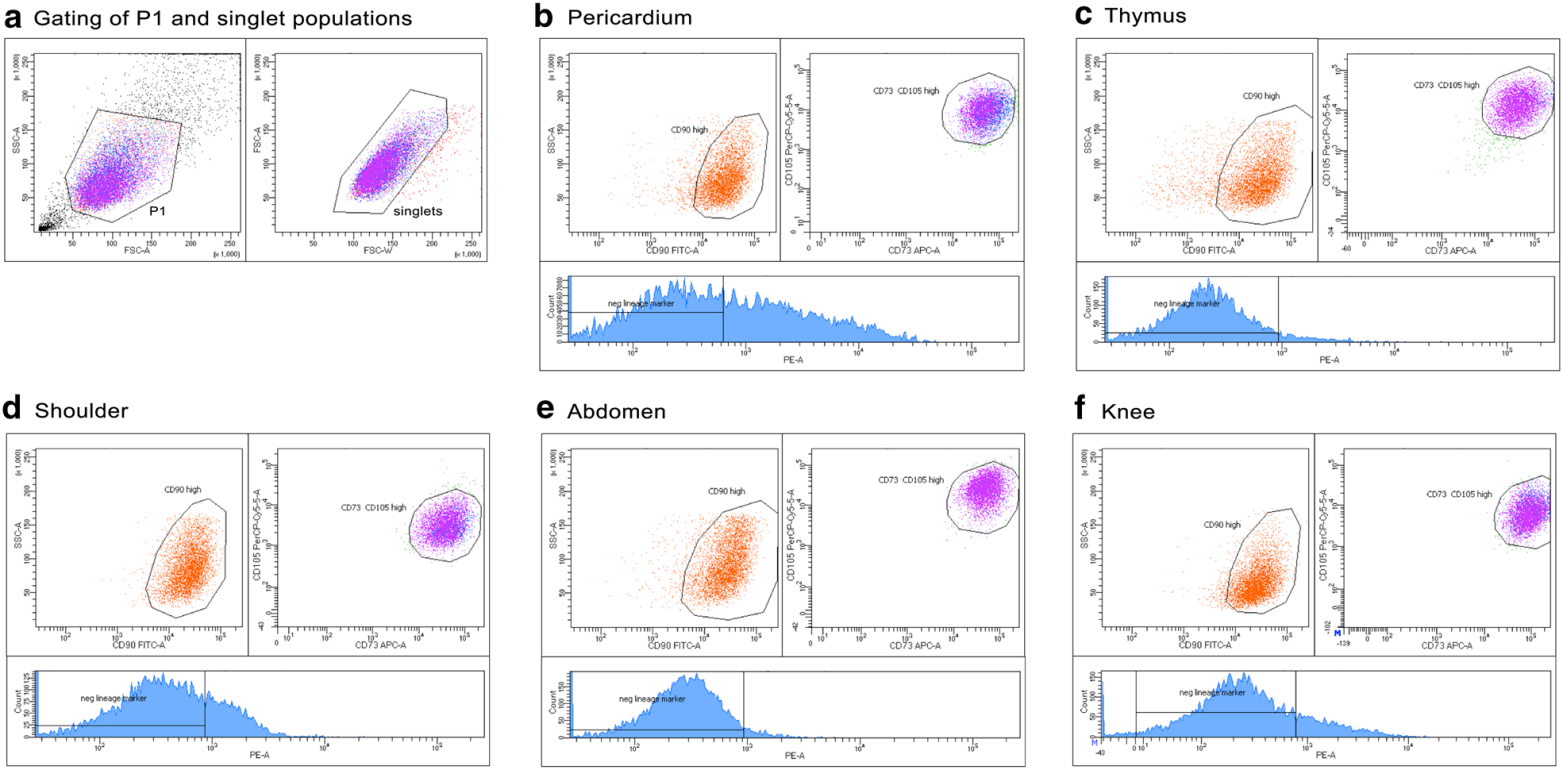
Flow cytometry characterization of isolated ASCs. Representative gating of P1 and singlet populations (**a**), and flow cytometry analysis for ASCs isolated from pericardium (**b**), thymus (**c**), shoulder (**d**), abdomen (**e**), and knee (**f**) (n = 3 for each location). The histogram for each tissue type depicts the negative lineage hematopoietic markers. For each respective anatomical location, the P1 population is positive for CD markers 90, 73, and 105. The histogram for each tissue type shows the negative lineage hematopoietic markers and HLA-DR, with the bars defined based on an unstained and isotype control. This characterization shows that the populations of isolated ASCs contain a majority of mesenchymal stem cells with limited contamination by hematopoietic cells

Following the gating of 10,000 viable cells as P1 (Fig. 1a), with an n = 3 for each anatomical adipose tissue location, doublet discrimination was performed to detect disproportions between cell size and cell signal in order to insure data collected was not skewed by cell aggregates. For each respective anatomical location, the P1 population is positive for CD markers 90, 73, and 105. Viable and singlet cells were marked for mesenchymal (CD90, CD73, and CD105) and hematopoietic markers (CD34, CD11B, CD19, and CD45), and HLA-DR. It was found that within this parent population that the majority of cells were positive for MSC markers CD90, CD73, and CD105, and negative for HSC markers (Mildmay-White and Khan 2016). This characterization shows that our populations of isolated ASCs are mesenchymal stem cells and are negative for hematopoietic cell surface markers. Specific statistical data for each population of cells is included in Additional file 1: Table S1.

### Bioenergetic profiling

**Fig. 2:**
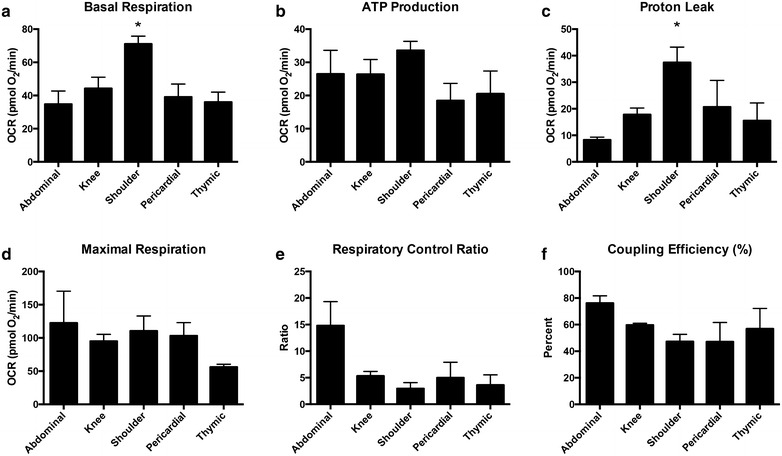
Mitochondrial stress test calculations. Values from each group of ASCs were averaged and then graphed together under the respective calculations made for: **a** basal respiration, **b** ATP production, **c** proton leak, **d** maximal respiration, **e** respiratory control ratio, and **f** coupling efficiency. Oxygen consumption rates (OCR) of shoulder ASCs were significantly higher than other ASC groups for both basal respiration and proton leak. Graphs are presented as mean ± SEM, with n = 4 for all organ specific adipose tissue types

Basal respiration of shoulder-region ASCs had the highest OCR when compared to the pericardial, thymic and abdominal groups (Fig. 2a). OCR associated with ATP production was similar across all groups with no significance (Fig. 2b). Proton leak OCR was significantly highest in shoulder ASCs compared to abdominal ASCs, and there were no significant differences between the other groups (Fig. 2c). Maximal respiration OCR was similar in all groups (Fig. 2d). Although the respiratory control ratio (RCR) was highest in abdominal ASC there were no significant differences between experimental groups (Fig. 2e). Similarly, there were no significant differences in coupling efficiency (CE) between groups (Fig. 2f).

**Fig. 3:**
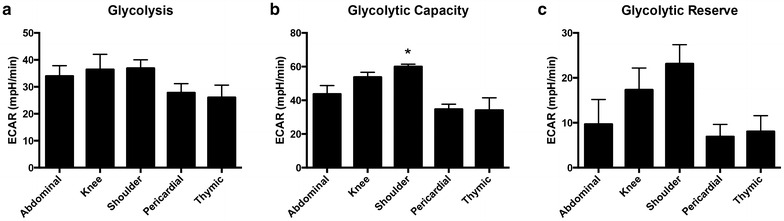
Glycolysis stress test calculations. Values from each group of ASCs were averaged and then graphed together under the respective calculations made for: **a** glycolysis, **b** glycolytic capacity, and **c** glycolytic reserve. Extracellular acidification rates (ECAR) of shoulder ASCs were significantly higher than other ASC groups for glycolytic capacity. Graphs are presented as mean ± SEM, with n = 4 for all organ specific adipose tissue types

There were no significant differences in glycolytic ECAR between the groups (Fig. 3a). The glycolytic capacity was significantly higher in shoulder ASCs than thymic and pericardial ASCs (Fig. 3b). There was no were no significant differences in glycolytic reserve between groups (Fig. 3c).

**Fig. 4:**
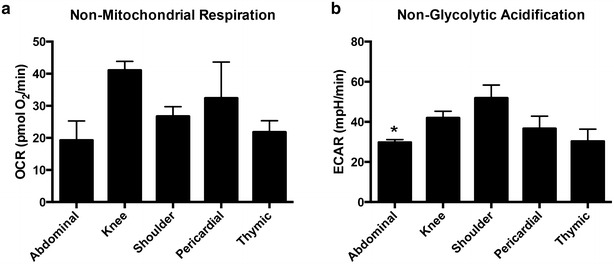
Non-mitochondrial respiration and non-glycolytic acidification calculations. No differences were appreciated in oxygen consumption rates (OCR) calculated for non-mitochondrial respiration (**a**). Abdominal extracellular acidification rates (ECAR) rates were lowest for ASCs under non-glycolytic acidification (**b**). Graphs are presented as mean ± SEM, with n = 4 for all organ specific adipose tissue types

Although non-mitochondrial respiration was highest in knee ASCs, there were no significant differences between the groups (Fig. 4a). ECAR for non-glycolytic acidification was significantly lower in abdominal ASCs compared to knee and shoulder ASCs (Fig. 4b).

## Discussion

The emerging field of regenerative medicine has identified adipose tissue as an abundant source of stem/stromal cells for tissue engineering applications. Due to its mesodermal origin, ASCs can differentiate into adipose lineage cells (Zuk et al. 2001), osteogenic cells (Gimble et al. 2011), chondrogenic cells (Ogawa et al. 2004) and myogenic cells (Bacou et al. 2004), making ASCs ideal for tissue repair and regeneration. In the present study, a basic flow cytometry panel was performed to confirm that a majority of cells isolated were positive for mesenchymal stem cell markers CD90, CD73, and CD105 (Fig. 1) and negative for hematopoietic markers, thereby supporting identification of ASCs despite anatomic location. Interestingly in previous research, ASCs isolated from subcutaneous tissue have been shown to demonstrate enhanced adipogenic differentiation capacity relative to ASCs derived from other areas (Russo et al. 2014). Numerous factors may contribute to the differentiation proficiency in these cells, including differences in reserve capacity as discussed below, changes in mtDNA (Hämäläinen 2016) or metabolic reconfiguration (Forni et al. 2016).

Extrapolating from this concept, we compared the bioenergetic profiles of adipose-derived stem/stromal cells isolated from five different subcutaneous adipose sites. The vascularization of tissues at different anatomical locations may affect and alter the cellular milieu and surrounding microenvironment, resulting in bioenergetic differences dependent on donor physiology. Indeed in both humans (Russo et al. 2014) and animals (Via et al. 2012; Rolfe and Brand 1997) there is adipose depot-dependent and donor-dependent variability that alters cell yield, viability, immunophenotype, doubling time, differentiation capacities, and metabolic differences. Overall, shoulder ASCs had the highest basal respiration, proton leak and glycolytic capacity. To discuss these parameters, basal respiration is defined as the oxygen consumed that is used to meet cellular ATP demand. Basal respiration is strongly controlled by ATP turnover, and partly by substrate oxidation and proton leak (Brand and Nicholls 2011). Proton leak refers to the remaining basal respiration not coupled to ATP production, and finally, glycolytic capacity refers to the part of ECAR used to meet cellular demands when ATP synthesis has been inhibited by oligomycin.

Conceptually, isolated mitochondria can reach the respiratory state 4o when ATP synthase is inhibited by oligomycin, stopping proton re-entry through the synthase and slowing respiration. Respiration is then strongly controlled by proton leak kinetics and partially by substrate oxidation in state 4o, making respiration responsive to dysfunction caused by uncoupling, relatively insensitive to changes in substrate oxidation, and completely insensitive to changes in ATP turnover (Brand and Nicholls 2011; Masini et al. 1983). State 4, which is similar to state 4o but with a contributing factor of ATP recycling, is sensitive to changes in both proton leak and ATP turnover (Divakaruni and Brand 2011). In these hypothetical states where the mitochondria are isolated, a large increase in proton leak would indicate that mitochondria are severely damaged due to uncoupling. In uncoupled states, there is also a propensity to have increased production of reactive oxygen species (ROS) and thereby increased tissue damage. However, proton leak is not merely an artifact of isolated mitochondria; it is also observed in intact cells. Indeed, proton leak can account for a large percentage of oxygen consumption in resting cells (Rolfe and Brand 1997; Divakaruni and Brand 2011; Krauss et al. 2002), though it should be noted that the proton leak data in the current experiment is unavoidably elevated since the addition of oligomycin inhibits phosphorylation and subsequently increases proton leak. In vivo, standard metabolic rate can vary between different major oxygen-consuming tissues. Cardiac tissue can have a low proton leak, whereas liver and skeletal muscle will have moderate and high proton leaks, respectively (Rolfe and Brand 1997; Rolfe and Brown 1997; Rolfe et al. 1999). Therefore, it might be expected that ASCs isolated from an area near skeletal muscle would have a higher proton leak value, since the ASCs within the shoulder adipose tissue contribute to healing and repair of shoulder muscles. Indeed in a rotator cuff model using ASCs, the adipose tissue and increased fatty infiltration of the muscle resulted in better healing and regeneration (Oh et al. 2014). Moreover, ASCs derived from rotator cuff regions have greater myogenic potential than bone marrow derived mesenchymal stem cells (Tsai et al. 2013; Valencia Mora et al. 2015). Since cellular ATP demand is also greater in skeletal muscle, it is logical for shoulder ASCs to have a higher basal respiration. Additionally, white adipose tissue can be classified into three major types based on stromal content, vascularity, and adipocyte morphology. Adipose tissue located in specific areas, including the shoulder, has been found to have moderate stroma and significant vascularization as compared to other types of white adipose tissue (Sbarbati et al. 2010). The increased vascular component supports the increased metabolic activity of ASCs residing in the shoulder.

In line with this logic, subcutaneous abdominal ASCs are isolated from a region that does not require fast cell turnover and cells that support an increased bioenergetic state, unlike ASCs from omental adipose tissue (Russo et al. 2014; Shah et al. 2014). In the present experiment, the ECAR of abdominal ASCs was found to be lowest of all groups under non-glycolytic acidification. Moreover, the higher glycolytic capacity observed in shoulder ASCs can be explained by the higher ECAR necessary to meet the cellular demands associated with increased basal respiration and proton leak.

In our studies we also estimated the coupling efficiency (CE) and the respiratory control ratio (RCR), of the isolated ASCs. CE is calculated as the proportion of mitochondrial respiratory rate used to drive ATP synthesis (i.e., perfectly coupled OXPHOS will have a CE = 100%, while a pure state 4 respiration will have CE = 0%) (Divakaruni and Brand 2011). RCR is calculated as a ratio between the cellular proton leak and their maximal capacity. Therefore, a change in either one of these two parameters will alter RCR, offering a solid estimation of overall bioenergetic dysfunction. Since CE and RCR are calculated as ratios, both measures are useful internal controls. However, the CE and RCR did not significantly differ between ASCs from each location in this study. The fact that we observed no significant differences in CE and RCR between groups could be explained by biomechanics and organ function. Due to the constant, but normal, wear and tear of pressures imposed upon the knee joints during ambulation, the cells in that peri-organ region require a faster rate of healing, proliferation, and regeneration. Similarly, the function of human heart requires the peri-organ milieu to support quick cell turnover, proliferation and regeneration. Furthermore, due to the high oxygen demand of cardiac cells required to sustain diastolic and systolic contractions, it is logical that pericardial ASCs would display a maximal respiration state compared to ASCs from skeletal muscle regions (e.g., shoulder).

## Conclusions

In summary, our data suggest that ASC utility is bioenergetically independent of anatomical adipose tissue harvest site. However, it is still likely that bioenergetic differences will vary depending on the individual “stem cell niche”, which describes the physiological microenvironment that supports stem cells in specific anatomic locations and regulates how stem cells participate in tissue generation, maintenance and repair (Via et al. 2012). Therefore, while subcutaneous ASC utility may be overall independent of the anatomical harvest site (Choudhery et al. 2015), the bioenergetic profile of ASCs from adipose tissue of various peri-organ regions will be reflected by its respective physiological functions, niche and microenvironment.

## Additional file

**Additional file 1: Table S1.** Data for each respective ASC population is shown as labeled. SD = standard deviation; P1 = population 1, which was gated for 10,000 viable cells.

## Abbreviations

ASCs: adipose-derived stem/stromal cells
SVF: stromal vascular fraction
MSCs: mesenchymal stem cells
OCR: oxygen consumption rates
OXPHOS: oxidative phosphorylation
RCR: respiratory control ratio
ECAR: extracellular acidification rate
NGA: non-glycolytic acidification
CE: coupling efficiency
ROS: reactive oxygen species

## References

[CR1] Aronowitz JA, Ellenhorn J (2013). Adipose stromal vascular fraction isolation: a head-to-head comparison of four commercial cell separation systems. Plast Reconstr Surg.

[CR2] Aronowitz JA, Lockhart RA, Hakakian CS, Hicok KC (2015). Clinical safety of stromal vascular fraction separation at the point of care. Ann Plast Surg.

[CR3] Bacou F, el Andalousi RB, Daussin PA, Micallef JP, Levin JM, Chammas M (2004). Transplantation of adipose tissue-derived stromal cells increases mass and functional capacity of damaged skeletal muscle. Cell Transplant.

[CR4] Baer PC, Geiger H (2012). Adipose-derived mesenchymal stromal/stem cells: tissue localization, characterization, and heterogeneity. Stem Cells Int.

[CR5] Brand MD, Nicholls DG (2011). Assessing mitochondrial dysfunction in cells. Biochem J.

[CR6] Choudhery MS, Badowski M, Muise A, Pierce J, Harris DT (2015). Subcutaneous adipose tissue-derived stem cell utility is independent of anatomical harvest site. Biores Open Access.

[CR7] Divakaruni AS, Brand MD (2011). The regulation and physiology of mitochondrial proton leak. Physiology (Bethesda).

[CR8] Estes BT, Diekman BO, Gimble JM, Guilak F (2010). Isolation of adipose-derived stem cells and their induction to a chondrogenic phenotype. Nat Protoc.

[CR9] Forni MF, Peloggia J, Trudeau K, Shirihai O, Kowaltowski AJ (2016). Murine mesenchymal stem cell commitment to differentiation is regulated by mitochondrial dynamics. Stem Cells.

[CR10] Gimble JM, Grayson W, Guilak F, Lopez MJ, Vunjak-Novakovic G (2011). Adipose tissue as a stem cell source for musculoskeletal regeneration. Front Biosci (Schol Ed).

[CR11] Hämäläinen RH (2016). Mitochondria and mtDNA integrity in stem cell function and differentiation. Curr Opin Genet Dev.

[CR12] Khalpey Z, Marsh KM, Ferng A, Bin Riaz I, Hemphill C, Johnson K (2015). First in man: sternal reconstruction with autologous stem cells. ASAIO J.

[CR13] Koźlik M, Wójcicki P (2014). The use of stem cells in plastic and reconstructive surgery. Adv Clin Exp Med.

[CR14] Krauss S, Zhang C-Y, Lowell BB (2002). A significant portion of mitochondrial proton leak in intact thymocytes depends on expression of UCP2. Proc Natl Acad Sci USA.

[CR15] Markarian CF, Frey GZ, Silveira MD, Chem EM, Milani AR, Ely PB (2014). Isolation of adipose-derived stem cells: a comparison among different methods. Biotechnol Lett.

[CR16] Masini A, Ceccarelli-Stanzani D, Muscatello U (1983). The effect of oligomycin on rat liver mitochondria respiring in state 4. FEBS Lett.

[CR17] McIntosh KR, Lopez MJ, Borneman JN, Spencer ND, Anderson PA, Gimble JM (2009). Immunogenicity of allogeneic adipose-derived stem cells in a rat spinal fusion model. Tissue Eng Part A.

[CR18] Mellor LF, Mohiti-Asli M, Williams J, Kannan A, Dent MR, Guilak F (2015). Extracellular calcium modulates chondrogenic and osteogenic differentiation of human adipose-derived stem cells: a novel approach for osteochondral tissue engineering using a single stem cell source. Tissue Eng Part A.

[CR19] Mildmay-White A, Khan W. Cell surface markers on adipose-derived stem cells: a systematic review. Curr Stem Cell Res Ther. 2016 10.2174/1574888X11666160429122133 27133085

[CR20] Ogawa R, Mizuno H, Hyakusoku H, Watanabe A, Migita M, Shimada T (2004). Chondrogenic and osteogenic differentiation of adipose-derived stem cells isolated from GFP transgenic mice. J Nippon Med Sch.

[CR21] Oh JH, Chung SW, Kim SH, Chung JY, Kim JY (2014). 2013 Neer Award: effect of the adipose-derived stem cell for the improvement of fatty degeneration and rotator cuff healing in rabbit model. J Shoulder Elbow Surg.

[CR22] Omar A, Chatterjee TK, Tang Y, Hui DY, Weintraub NL (2014). Proinflammatory phenotype of perivascular adipocytes. Arterioscler Thromb Vasc Biol.

[CR23] Pikuła M, Marek-Trzonkowska N, Wardowska A, Renkielska A, Trzonkowski P (2013). Adipose tissue-derived stem cells in clinical applications. Expert Opin Biol Ther.

[CR24] Rolfe DF, Brand MD (1997). The physiological significance of mitochondrial proton leak in animal cells and tissues. Biosci Rep.

[CR25] Rolfe DF, Brown GC (1997). Cellular energy utilization and molecular origin of standard metabolic rate in mammals. Physiol Rev.

[CR26] Rolfe D, Newman J, Buckingham JA, Clark MG, Brand MD (1999). Contribution of mitochondrial proton leak to respiration rate in working skeletal muscle and liver and to SMR. Am J Physiol.

[CR27] Russo V, Yu C, Belliveau P, Hamilton A, Flynn LE (2014). Comparison of human adipose-derived stem cells isolated from subcutaneous, omental, and intrathoracic adipose tissue depots for regenerative applications. Stem Cells Transl Med.

[CR28] Sachs PC, Francis MP, Zhao M, Brumelle J, Rao RR, Elmore LW (2012). Defining essential stem cell characteristics in adipose-derived stromal cells extracted from distinct anatomical sites. Cell Tissue Res.

[CR29] Sbarbati A, Accorsi D, Benati D, Marchetti L, Orsini G, Rigotti G (2010). Subcutaneous adipose tissue classification. Eur J Histochem.

[CR30] Schwartz B, Yehuda-Shnaidman E (2014). Putative role of adipose tissue in growth and metabolism of colon cancer cells. Front Oncol.

[CR31] Shah FS, Wu X, Dietrich M, Rood J, Gimble JM (2013). A non-enzymatic method for isolating human adipose tissue-derived stromal stem cells. Cytotherapy.

[CR32] Shah FS, Li J, Dietrich M, Wu X, Hausmann MG, LeBlanc KA (2014). Comparison of stromal/stem cells isolated from human omental and subcutaneous adipose depots: differentiation and immunophenotypic characterization. Cells Tissues Organs (Print).

[CR33] Shin S-H, Yun TK, Han S-K, Jeong S-H, Dhong E-S, Kim W-K (2015). Comparison of the matrix synthesizing abilities of human adipose-derived stromal vascular fraction cells and fibroblasts. J Craniofac Surg.

[CR34] Tsai C-C, Huang T-F, Ma H-L, Chiang E-R, Hung S-C (2013). Isolation of mesenchymal stem cells from shoulder rotator cuff: a potential source for muscle and tendon repair. Cell Transpl.

[CR35] Valencia Mora M, Ruiz Ibán MA, Díaz Heredia J, Barco Laakso R, Cuéllar R, García Arranz M (2015). Stem cell therapy in the management of shoulder rotator cuff disorders. World J Stem Cells.

[CR36] Via AG, Frizziero A, Oliva F (2012). Biological properties of mesenchymal Stem Cells from different sources. Muscles Ligaments Tendons J.

[CR37] Zuk PA, Zhu M, Mizuno H, Huang J, Futrell JW, Katz AJ (2001). Multilineage cells from human adipose tissue: implications for cell-based therapies. Tissue Eng.

