# Supplementary figures and images for: Adipose-derived human stem/stromal cells: comparative organ specific mitochondrial bioenergy profiles
Source: Springerplus. 2016 Dec 1;5(1):2057. doi: 10.1186/s40064-016-3712-1 (PMC5133220; doi:10.1186/s40064-016-3712-1)

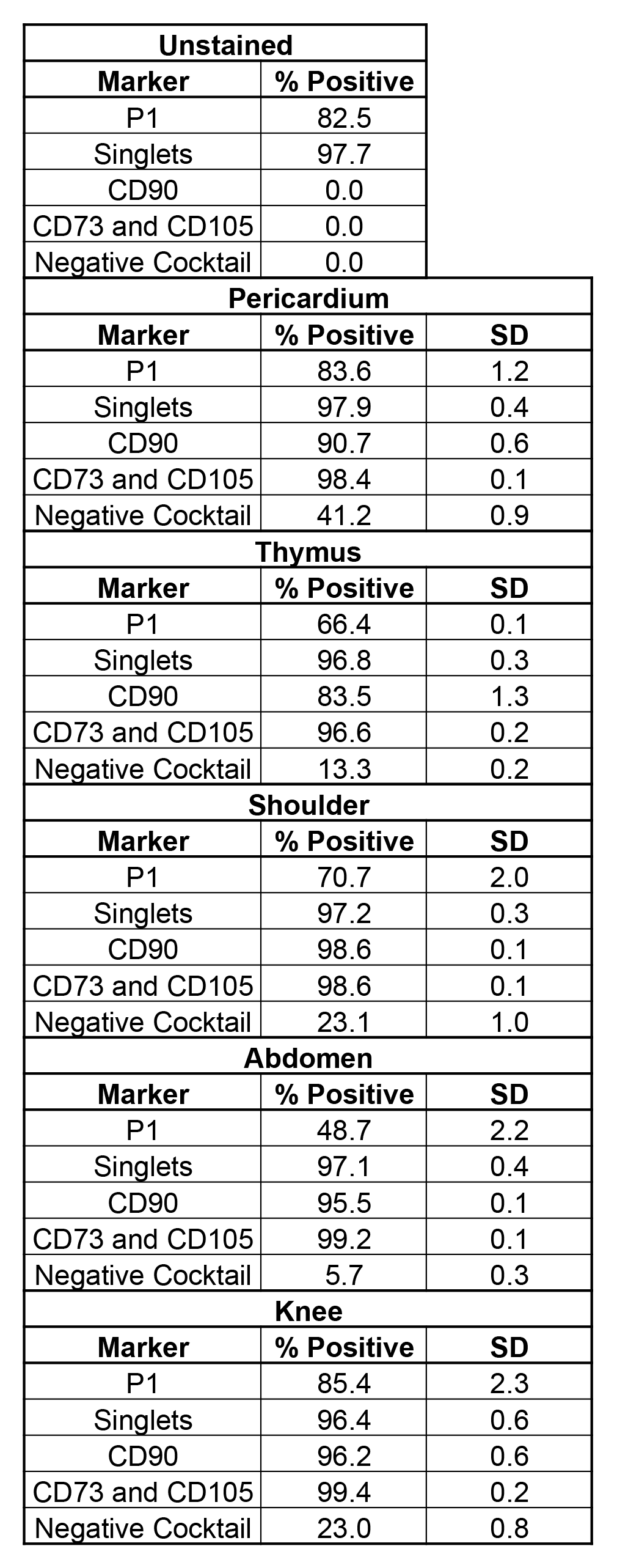

Supplement: Supplementary file 1 — Additional file 1: Table S1. Data for each respective ASC population is shown as labeled. SD = standard deviation; P1 = population 1, which was gated for 10,000 viable cells. [file 40064_2016_3712_MOESM1_ESM.tif]
